# Incidence and determinants of mortality among adult HIV infected patients on second-line antiretroviral treatment in Amhara region, Ethiopia: a retrospective follow up study

**DOI:** 10.11604/pamj.2019.33.89.16626

**Published:** 2019-06-06

**Authors:** Adino Tesfahun Tsegaye, Wagaye Alemu, Tadesse Awoke Ayele

**Affiliations:** 1 Department of Epidemiology and Biostatistics, University of Gondar, College of Medicine and Health Sciences, Institute of Public Health, Gondar, Ethiopia; 2 Department of Epidemiology and Biostatistics Ethiopia, Dilla University, College of Medicine and Health Sciences, Dilla, Ethiopia

**Keywords:** ART, HIV, Ethiopia, Mortality, second-line

## Abstract

**Introduction:**

Mortality of adult patients who are on antiretroviral therapy (ART) is higher in low-income than in high-income countries. After the failure of standard first-line treatment, patients switch to second-line regimens. However, there are limited data about the outcome of patients after switching to a second-line regimen in the study area. This study aimed to measure the rate of mortality and its determinants among HIV patients on second-line ART regimens.

**Methods:**

Multicenter institution based retrospective follow up study was conducted among 1192 adult patients who started second-line ART between 2008 and 2016 in eight selected hospitals of Amhara region. Patients who started second-line treatment after the failure of first-line treatment were included. Patient medical records, registration books, and computer database were used to collect the data. Time to death after a switch to second-line ART was the primary outcome of interest. Cox proportional hazard model was fitted to identify determinant factors of mortality.

**Results:**

Among 1192 patients who were on second-line ART, 136 (11.4%) died with 3,157 person-years of follow up. Over the study period, the mortality rate was 4.33 per 100 person-years. Not taking isoniazid preventive therapy (IPT) (Adjusted Hazard Ratio (AHR): 6.6; 95% CI: 2.9, 15.0), did not make modification on second-line regimen (AHR: 4.4; 95% CI: 2.8, 6.8), poor clinical adherence (AHR: 2.5; 95% CI: 1.4, 4.5), functional status of bedridden (AHR: 2.7; 95% CI: 1.5, 4.8), and having attained a tertiary level of education (AHR: 0.4; 95% CI: 0.2, 0.8) were independent determinants of mortality.

**Conclusion:**

The incidence rate of mortality was high and most of the deaths occurred within 12 months after switching to second-line ART. Higher mortality among adult HIV-infected patients was associated with poor adherence, no formal education, not taking IPT, being bedridden at the time of the switch, and not modifying second-line treatment. Improving treatment adherence of patients by providing consistent adherence counseling, providing INH prophylaxis and monitoring patient's regimen more closely during the first twelve months after switch could decrease mortality of HIV patients on a second-line regimen.

## Introduction

Acquired Immune Deficiency Syndrome (AIDS), which is caused by the Human Immunodeficiency Virus (HIV), has been the major public health problem worldwide [1]. The majority of HIV infected individuals live in sub-Saharan Africa (SSA) [2]. In these countries, AIDS-related morbidity and mortality remain the highest in the world because of limited access to HIV diagnosis and treatment. According to the 2012 UNAIDS report, around 1.7 million people died from AIDS-related causes worldwide, and 70% occurred in SSA [3]. Ethiopia is one of the hardest hit sub-Saharan African countries by the HIV pandemic with an estimated death of 52,405 by 2014 [4]. Since the introduction of Zidovudine (ZDV) in 1987 as a first antiretroviral drug, there have been significant advancements in the antiretroviral treatment [5]. Currently, there are six classes of antiretroviral drugs such as Nucleoside reverse transcriptase inhibitors (NRTIs), Non-nucleoside reverse transcriptase inhibitors (NNRTIs), Protease inhibitors (PIs), Integrase inhibitors (IIs), Fusion inhibitors (FIs), and Chemokine receptor antagonists (CRAs) [5]. For a new patient who is going to start ART, a combination of three drugs is provided. The drug combination contains two NRTIs and one NNRTIs [6]. After highly active antiretroviral treatment (HAART) introduced in 1995-96, HIV infection turns from inevitable fatal condition into chronic manageable disease [7]. In recent years, efforts have been made to expand access to ART in low-income countries, and it is showing encouraging results [8-11]. The benefit of ART in restoring immune function and reducing HIV related morbidity and mortality is lost when a patient develops treatment failure [12, 13]. This happens when a patient has poor adherence, drug resistance, high baseline plasma viral load, and low baseline CD4 count [14-19]. Following a failure of the first-line regimen, patients switch to a second-line regimen containing two new NRTIs and one PI [6, 20, 21]. Since third-line regimens are costly and not readily available in resource-limited countries, second-line regimens are often the last therapeutic option available for patients in these settings [22]. Not all patients who initiate antiretroviral therapy respond well. Some patients may not respond due to poor adherence, suboptimal antiretroviral treatment potency, and genetic mutation of HIV strains [23]; this could be followed by death. Since living longer with HIV is one of the global strategies, provision of ART by itself is not enough to control the problems of HIV treatment; rather its therapeutic effect on improving survival needs thorough monitoring and evaluation through scientific research. Though some studies based in Ethiopia investigate the outcomes of first-line HIV treatment; little has been done regarding second-line treatment. Therefore this study aimed to measure the incidence and determinants of mortality after a switch to second-line ART regimens.

## Methods

**Study design and setting:** a multi-center institution-based retrospective follow-up study was conducted in eight selected governmental hospital found in Amhara regional state, Ethiopia. Five referral hospitals (Dessie, Bahirdar (Felegehiwot), Debremarkos, University of Gondar, and Debre Birhan Referral Hospitals) and three general hospitals (Finote Selam, Woldiya, and Debretabor General Hospitals) were included in this study. The hospitals were selected purposively based on their representativeness to the people of the Amhara regional state. Almost all bigger hospitals which provide second-line ART in the region were included. All of the hospitals initiated ART in 2005. By 2014, there were more than 220,000 people who ever enrolled in HIV care and 102,088 current ART users in the region. Among the ART users, 1.5% of them were on second-line ART [24]. In accordance with the current recommendation, reverse transcriptase inhibitors (NRTI) such as lamivudine (3TC), zidovudine (AZT), and tenofovir (TDF); non-nucleoside reverse transcriptase inhibitors (NNRTI) such as efavirenz (EFV), nevirapine (NVP); and protease inhibitors (PI) such as atazanavir/ritonavir (ATV/r), lopinavir/ritonavir (LPV/r) were used. For first-line treatment, a combination of two NRTIs with one NNRTI, and for second-line treatment two NRTIs with one PI [25, 26] was used. At each visit, patients' clinical condition including the WHO clinical stage, functional status, weight, and medication adherence were assessed by the providers. The CD4 count was measured at baseline and every six months or when indicated. According to the current practice, if the patients develop treatment failure of first-line ART (clinical, immunological, or virological), they switch to second-line ART.

**Participants:** the study population was adult HIV patients aged above 15 years who started second-line ART in the selected hospitals between February 2008 and January 2016. The data were extracted from February to April 2016. Patients who initiated second-line therapy in the selected hospitals with a documented failure of first-line therapy were included, and patients who transferred in after started second-line treatment in other places were excluded. Initially, the calculated sample size was 1024. However, from our preliminary assessment, we found 1233 patients who fulfilled our inclusion criteria and we have included all of them in the study. In the actual data collection, 41 of them had an incomplete record which couldn't able us to entertain their outcome and they were excluded in the final model. Finally, 1192 patient records were used for the final analysis. Data were collected from medical records, registers, and computer database. Twenty trained ART nurses collected the data using a pretested data collection checklist under the supervision of 5 public health professionals who had a Master of Public Health (MPH). The collected data were checked on a daily bases for consistency, completeness, and accuracy; appropriate corrections were made.

**Variables of the study:** the primary outcome variable was time to death. It was defined as the death of a patient while on second-line ART. Its incidence was measured by using person-time at risk starting from the time of switch to a second-line regimen until each patient ended follow-up. The independent variables were socio-demographic factors (sex, age, educational status, environmental factors (health facility), occupation) and clinical and treatment-related factors (body mass index, CD4 count, CPT, IPT, functional status, WHO clinical stage, opportunistic infections, presence of severe side effects, presence of active TB, adherence, ART drug regimen, duration of treatment, regimen modification). Transferred out refers to patients who are transferred to other health care facilities. A loss to follow-up (LTFU) was defined as a patient who did not receive ART refills for a period of three months or longer and is not yet classified as “dead” or “transferred-out”. Patients were considered censored if they transferred out, lost to follow up or remain on follow up at the end of the study. The clinical adherence of participants was assessed and it was defined as a regular attendance of patients according to a given appointment. If the patients were coming regularly by the given appointment dates in >85% of the times, they were considered as having good clinical adherence, and <85% attendance as poor clinical adherence [25, 26].

**Statistical analysis:** the collected data were entered using EPI-INFO version 7.00 and then exported to STATA version 12.0 for further analysis. A life table was used to estimate the cumulative survival of patients; Kaplan Meier failure curve was used to estimate mortality rate, and a log-rank test was used to compare failure curves between the different categories of the explanatory variables. Schoenfeld residuals test (both global and scaled) and graphical methods were used to check Cox proportional hazards assumption. The P-value for the global test was 0.1 (not significant), which indicates the model was fit. Cox proportional hazards model was used to identify determinants of mortality. Variables having a p-value of 0.2 or less in the bi-variable analysis were fitted into the multivariable model. Ninety-five percent confidence interval of hazard ratio (HR) was computed and variables having a p-value less than 0.05 in the multivariable Cox proportional hazards model were considered as statistically significant.

**Ethics consideration:** ethical clearance was obtained from the institutional review board (IRB) of the University of Gondar. A letter of support and a permission letter were obtained from the Amhara Regional State Health Bureau and the hospital's administration respectively. All patient records were de-identified and kept locked. Since we were reviewed patient records, informed consent was waived.

## Results

**Baseline characteristics of the study participants:** out of 1192 samples included in the final analysis, 387 (32.5 %) were from Dessie referral hospital followed by Felegehiwot referral hospital (378 (31.7 %)). The median age of patients at the start of antiretroviral treatment was 32 years with an Interquartile range (IQR) of 11 years, and the majority of the patients 609 (51.1 %) were males (Table 1). With regard to their duration of stay on first-line ART, 348 (29.2 %) of patients stayed 12 to 36 months, and 101 (8.5 %) of patients stayed less than 12 months. Before switching to a second-line regimen, regimens were modified for 535 (43.85 %) participants and among them, it was modified only once for 423 (79.07%), two times for 100 (18.69%) and three times and above for 12 (2.24%) patients. Majority of the patients (541 (46.24%)) switched to second-line ART regimen because of immunological failure of first-line treatment (Table 1). Regarding the regimen they take, 406 (33.17%) used TDF_3TC_LPV/r and 201(16.47%) of patients used the drug combination ABC-ddi-LPV/r (Table 2). The majority (929 (77.9 %)) had a clinical adherence level of < 85%; 379 (31.8 %) had a base-line CD4 count of <=50 cells/mm^3^; 843 (70.7 %) were given cotrimoxazole; and 277 (23.2 %) received isoniazid (INH) preventive therapies.

**Table 1:** Baseline characteristics of adult HIV patients on second-line ART in Amhara region February 2008- 2016

Variable	Frequency	Percent (%)
**Age**		
15-29	425	35.7
30-39	514	43.1
40-49	196	16.4
>=50	57	4.8
**Sex**		
Female	583	48.9
Male	609	51.1
**Educational status**		
No formal education	392	32.9
Primary	239	20.1
Secondary	407	34.1
Tertiary	154	12.9
**Facility/Hospital**		
University of Gondar	140	11.7
Debre Birhan hospital	22	1.9
Dessie RH	387	32.5
Debre Tabor hospital	24	2.0
Felegehiwot RH	378	31.7
Debre Markos	47	3.9
Woldiya	160	13.4
Finote Selam Hospital	34	2.9
**Baseline CD4 count (cells/mm^3^)**		
<=50	379	30.7
51-200	612	49.6
>200	201	16.3
**Reason for switch**		
Clinical failure	334	28.0
Immunological failure	563	47.2
Virological failure	295	24.7
**WHO clinical stage (T stage) at the switch**		
Stage1	636	53.4
Stage2	119	10.0
Stage3	356	29.9
Stage4	81	6.8
**CPT given**		
No	349	29.3
Yes	843	70.7
**INH Given**		
No	915	76.8
Yes	277	23.2
**Baseline functional status**		
Working	745	62.5
Ambulatory	389	32.6
Bedridden	58	4.9
**NRTI Backbone at the switch**		
TDF	534	44.8
AZT	239	20.1
ABC	398	33.4
D4t & DDI	21	1.8
**PI Drugs**		
LPV/R	837	70.2
ATV/R	349	29.3
NFV/R	6	0.5

CPT, cotrimoxazole preventive therapy; d4t, stavudine; ddi, didanosine; INH, isoniazid;

**Table 2:** Life table for the probability of death of adult HIV patients on second-line ART in Amhara region, Ethiopia, February 2008-2016

Interval	Total	Deaths	Lost	Cumulative probability of death [95% Conf. Int.]	
0-6	1192	13	99	0.01	(0.01-0.02)
6-12	1080	54	136	0.06	(0.05-0.08)
12-18	890	26	112	0.09	(0.08-0.11)
18-24	752	8	116	0.10	(0.90-0.12)
24-30	628	6	98	0.11	(0.09-0.14)
30-36	524	3	81	0.12	(0.09-0.14)
36-42	440	2	84	0.12	(0.10-0.15)
42-48	354	2	64	0.13	(0.11-0.15)
48-54	288	2	59	0.14	(0.11-0.16)
54-60	227	3	43	0.15	(0.12-0.18)
60-66	181	3	55	0.16	(0.13-0.20)
66-72	123	0	50	0.16	(0.13-0.20)
72-78	73	1	36	0.18	(0.14-0.23)
78-84	36	0	19	0.18	(0.14-0.23)
84-90	17	0	7	0.18	(0.14-0.23)
90-96	10	0	10	0.18	(0.14-0.23)

**Figure 1:**
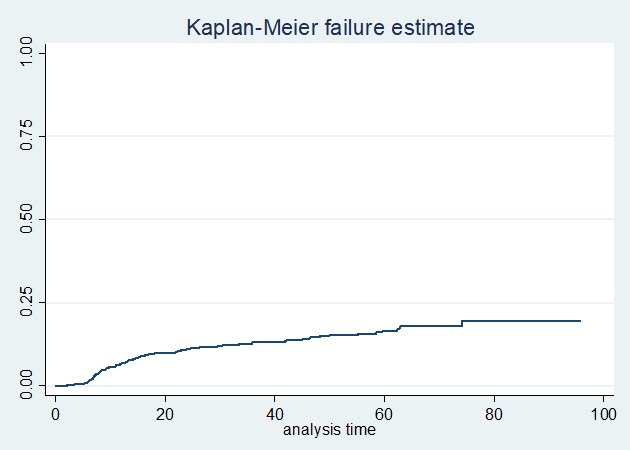
Kaplan Meier failure curve of mortality on second-line ART among adult HIV patients in Amhara region, Ethiopia February 2008-2016

**Figure 2:**
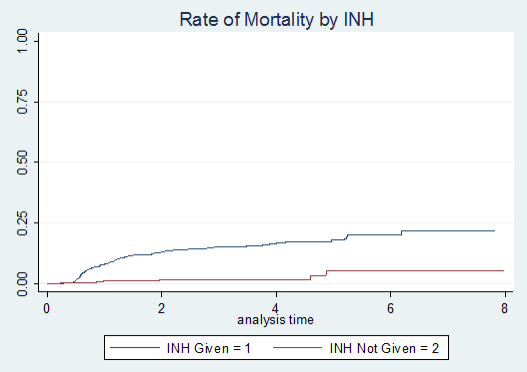
Kaplan Meier failure curve of mortality on second-line ART among adult HIV patients in Amhara region, Ethiopia February 2008-2016 based on isoniazid prophylaxis

**Figure 3:**
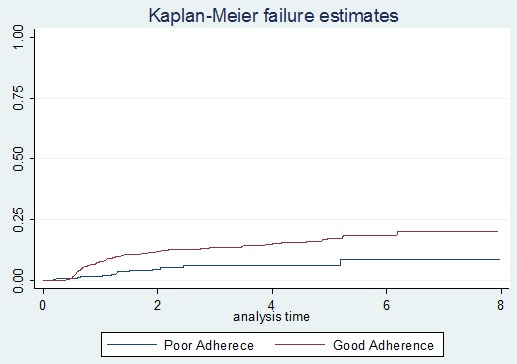
Kaplan Meier failure curve of mortality on second-line ART among adult HIV patients in Amhara region, Ethiopia February 2008-2016 based on clinical adherence

**Mortality after switching to second-line ART:** among 1192 adult HIV-infected patients on second-line ART, 136 (11.41%) died; out of them, 56 (41.17%) died within 12 months after switching to second-line treatment. The mean follow-up time of patients on first-line ART was 49.51 months (SD ± 28.18). The median follow-up time on second-line ART was 25.3 months (IQR 34.66). The study cohort had contributed a total of 3157 person-years of follow-up. Over the study period, the total mortality rate was 4.33 per 100 person-years. The mortality rate of patients at the end of the 12^th^ month after switching to second-line ART was 39.6 per 1000 person-years, 24 per 1000 person-years at 36^th^ month, 8 per 1000 person-years at the 72^nd^ month, and 2 per 1000 person-years at 96^th^ month (Figure 1). The incidence of mortality among INH users was 7.4 per 1000 person-years and among non-users was 51.3 per 1000 person-years (p-value <0.000) (Figure 2). The incidence of mortality in patients who had good clinical adherence was 18 per 1000 person-years and it was 50 per 1000 person-years in those who had poor clinical adherence (p-value = 0.0003) (Figure 3).

**Factors associated with mortality of HIV positive patients on second-line ART:** in the bivariable Cox proportional hazard analysis; educational status, baseline functional status, WHO clinical stage at the switch, INH prophylaxis, the reason of eligibility to ART, second-line regimen modification, and adherence were statistically significant. Variables such as age, baseline CD4 count, facility type, the reason of switch to a second-line regimen, gender, occupation, baseline BMI, and past TB treatment had a P-value of >0.2 in the bivariable model and were not included in the multivariable model. In the multivariable Cox regression analysis, the independent predictors of mortality were not taking INH preventive therapy (AHR: 6.9; 95% CI: (3.0, 15.7)), did not make modification on second-line regimen (AHR: 4.8; 95% CI: (3.1, 7.5)), poor clinical adherence (AHR: 2.6; 95% CI: (1.4, 4.6)), functional status of bedridden (AHR: 3.0; 95CI (1.7, 5.5)), and having attained of tertiary education (AHR: 0.4; 95%CI: (0.2, 0.9)) (Table 3). The second-line regimen modification, tertiary education, functional status of working, good adherence and using INH prophylaxis had protective effects on mortality of patients. The risk of death was 4.8 times higher for those patients with second-line regimen was not modified as compared to those with second-line regimen modified. The rate of mortality was 3 times higher among those who were bedridden at the switch to a second-line regimen compared with those who were working. The risk of death was 2.6 times higher for patients who had poor clinical adherence compared with those with good clinical adherence. Patients who had tertiary education were 60% less likely to die than those patients who had no formal education. Patients who did not take INH were 6.9 times at higher risk of death compared with those who did take INH.

**Table 3:** Bivariable and multivariable Cox regression analysis of predictors of mortality on second-line ART among adult HIV patients in Amhara region, Ethiopia, February 2008-2016

Variable	Survival status		Crude HR(95%CI)	Adjusted HR(95%CI)
	Event	Censored		
*Educational status				
No education	378(88.9)	47(11.1)	1	1
Primary	465(90.5)	49(9.5)	0.6(0.4-1.0)	0.7(0.4-1.2)
Secondary	179(91.3)	17(8.7)	0 .9(0.7-1.4)	0.9(0.7-1.5)
Tertiary	47(82.5)	10(17.5)	0.4(0.2-0.8)	0.4(0.2-0.9)
*INH given				
No	798(87.2)	117(12.8)	6.6(2.9-15.0)	6.9(3.0-15.7)
Yes	271(97.8)	6(2.2)	1	1
*Second-line regimen modification				
No	565(85.2)	98(14.8)	4.4(2.8-6.8)	4.8(3.1-7.5)
Yes	504(95.3)	25(4.7)	1	1
*Clinical adherence level				
>=85%	250(95.1)	13(4.9)	1	1
<85%	819(88.2)	110(11.8)	2.5(1.4-4.5)	2.6(1.4-4.6 )
*Functional status				
Working	677(90.9)	68(9.1)	1	1
Ambulatory	348(89.5)	41(10.5)	0.9(0.7-1.5)	1.2(0.8-1.8)
Bedridden	44(75.9)	14(24.1)	2.7(1.5-4.8)	3.0(1.7-5.5 )
**WHO clinical stage at switch**				
Stage1	572(89.9)	64(10.1)	1	1
Stage 2	102(85.7)	17(14.3)	1.4(0.8-2.3)	1.3(0.7-2.2)
Stage 3	324(91.0)	32(9.0)	0.8(0.5-1.2)	0.7(0.6-1.2)
Stage 4	71(87.7)	10(12.3)	1.1(0.6-2.1)	0.9(0.5-1.9)

Event=death-censored=transfer out +lost to follow up+ alive (on treatment at the end of the study)

* Significant factors

## Discussion

This study aimed to measure mortality and its determinants among HIV infected adult patients on second-line antiretroviral therapy. The incidence of mortality was 4.33 per 100 person-years; which is consistent with studies done in India, in resource-limited countries and in sub-Saharan African countries [27-29]. However, it is higher than studies done in Zambia [30] and Switzerland [31]. The higher rate of mortality in this study might be due to the fact that most patients had advanced disease at baseline (CD4 count ≤200 cells/mm^3^); that could lead them to have bad outcomes [29]. The delay to switch to second-line regimens, that usually happens in resource-limited settings, might explain the higher mortality [32]. Different factors have been identified as determinants of mortality of patients on second-line ART. In the multivariable analysis: INH, second-line regimen modification, clinical adherence, functional, and educational status were found statistically significant determinants of mortality. Patients who had a history of second-line regimen modification after the switch had a lower risk of death than those had not. This is similar to a study done in resource-limited countries [28]. Patients who had modified their regimen might get better follow up and get their problems managed early. In addition, since deferring a drug modification while it is required might have a negative implication of accumulation of drug resistance [33], this group of patients might be benefited by the regiment modification which could help them to overcome an emerging drug resistance that could be a potential cause for lower immunity and death. Having good clinical adherence was protective of mortality. This evidence is supported by previously done researches [34, 35]. Having good clinical adherence, patients wouldn't discontinue their drugs, have close follow up and counseling form the health care providers, and they might be also psychologically stable to take their treatment. Due to these reasons, bad outcomes are less likely to happen among these patients. Taking INH had a protective effect on mortality of patients. This is similar to a study done in resource-limited countries, and Ethiopia [28, 35]. As already known, TB is the commonest opportunistic infection (OI) among HIV patients and it is a potential cause of death. If an HIV patient takes INH prophylaxis, it would prevent the morbidity and mortality due to TB. Patients who were bedridden at switch were at higher risk of death. This is consistent with a finding from eastern Ethiopia [36]. Functional status of patients has a correlation with their clinical and immunological status. Usually, patients in an advanced stage of the disease are bedridden and the chance of death is higher among those people [34]. Patients who were illiterate had a high risk of mortality compared to those with tertiary education. This is similar to a study done in Addis Ababa, Somalia and Tigray regions Ethiopia [35, 37, 38]. This could be due to the reason that educated people follow their treatment appropriately and may have better health-seeking behavior for different OIs and other related diseases. The main strength of this study is its representativeness in terms of covering multiple treatment centers, and the length of follow up time which was long enough to estimate mortality. However, it has its own limitations. Considering all death as HIV related could overestimate the incidence of mortality. On the contrary, the lost to follow up patients might include individuals who died at home without being reported and that could underestimate the incidence of mortality. In addition, residual confounding could present by the absence of viral load record, under-reporting of clinical conditions, and missing laboratory results. The data were collected in a retrospective fashion using secondary sources, with resulting incompleteness.

## Conclusion

The incidence rate of mortality was high and most of the deaths occurred within 12 months after switching to second-line ART. Higher mortality among adult HIV-infected patients was associated with poor adherence, no formal education, not taking IPT, being bedridden at the time of the switch, and not modifying second-line treatment. Improving treatment adherence of patients by providing consistent adherence counseling, providing INH prophylaxis and monitoring patient’s regimen more closely during the first twelve months after switch could decrease mortality of HIV patients on a second-line regimen.

### What is known about this topic

- Following the failure of the first-line regimen, patients switch to a second-line regimen;
- Third-line regimens are costly and not readily available in resource-limited countries;
- Not all patients who initiate antiretroviral therapy respond well.

### What this study adds

- Mortality after a switch to second-line regimens is high during the first twelve months after the switch;
- Getting INH prophylaxis, having good clinical adherence, attaining a tertiary level of education, and making regimen modification after a switch to second-line regimens are the factors which could decrease the rate of mortality among HIV patients on second-line ART;
- Close monitoring of patients after the switch to second-line ART could make the treatment outcome better.

## Competing interests

The authors declare no competing interests.
